# Endoscopic vacuum therapy for in- and outpatient treatment of colorectal defects

**DOI:** 10.1007/s00464-020-08172-5

**Published:** 2020-12-01

**Authors:** Florian Kühn, Ulrich Wirth, Julia Zimmermann, Nicola Beger, Sandro M. Hasenhütl, Moritz Drefs, Christian Heiliger, Maria Burian, Jens Werner, Tobias S. Schiergens

**Affiliations:** Department of General, Visceral, and Transplant Surgery, Ludwig-Maximilians-University Hospital, Campus Grosshadern, Marchioninistr. 15, 81377 Munich, Germany

**Keywords:** Endoscopic vacuum therapy, Colorectal defects, Anastomotic leakage, Complication management, Outpatient treatment

## Abstract

**Background:**

Evidence for endoscopic vacuum therapy (EVT) for colorectal defects is still based on small patient series from various institutions, employing different treatment algorithms and methods. As EVT was invented at our institution 20 years ago, the aim was to report the efficacy and safety of EVT for colorectal defects as well as to analyze factors associated with efficacy, therapy duration, and outpatient treatment.

**Methods:**

Cohort study with analysis of prospectively collected data of patients receiving EVT for colorectal defects at a tertiary referral center in Germany (*n* = 281).

**Results:**

The majority of patients had malignant disease (83%) and an American Society of Anesthesiologists classification of III/IV (81%). Most frequent indications for EVT were anastomotic leakage after sigmoid or rectal resection (67%) followed by rectal stump leakage (20%). EVT was successful in 256 out of 281 patients (91%). EVT following multi-visceral resection (*P* = 0.037) and recent surgical revision after primary surgery (*P* = 0.009) were risk factors for EVT failure. EVT-associated adverse events occurred in 27 patients (10%). Median treatment duration was 25 days. Previous chemo-radiation (*P* = 0.006) was associated with a significant longer duration of EVT. Outpatient treatment was conducted in 49% of patients with a median hospital stay reduction of 15 days and 98% treatment success. Younger patient age (*P* = 0.044) was associated with the possibility of outpatient treatment. Restoration of intestinal continuity was achieved in 60% of patients where technically possible with a 12-month rate of 52%.

**Conclusions:**

In patients with colorectal defects, EVT appears to be a safe and effective, minimally invasive option for in- and outpatient treatment.

In recent years, the principle working mechanisms of vacuum-assisted wound therapy have been successfully applied for endoscopic treatment of various upper and lower gastrointestinal defects [1–3]. Active drainage of an infectious focus via an open-pored polyurethane sponge leads to a decrease in bacterial contamination, secretion, and local edema, while also promoting perfusion and granulation [4]. Endoscopic vacuum therapy (EVT) has been introduced for the management of gastrointestinal perforations and postoperative defects. EVT for anastomotic leakage after rectal resection was developed and implemented in clinical routine at our institution at the turn of the century. In 2007, first results on successful EVT in 28 out of 29 patients with anastomotic leakage after rectal resection were published by our institution [1]. Today, EVT is the most commonly used technique for endoscopic treatment of postoperative surgical leaks. Commercial systems for EVT are distributed in more than 40 countries worldwide [5, 6]. However, existing evidence on EVT for colorectal defects is still based on a few, small patient series from various institutions, employing different methods, treatment algorithms and materials [7]. In a recent review comparing 17 studies with 276 patients overall, success rates of EVT for treatment of anastomotic leakage after rectal resection range between 54 and 96% [7]. Treatment duration in the current literature varies between 11 and 244 days [7]. Hence, the duration of treatment is one of the major concerns. However, there is still no reliable data on EVT as an ambulatory treatment option and no evidence on outpatient treatment. As EVT was invented and implemented into clinical routine at our institution around 20 years ago, we have a broad experience on this technique. As high-volume colorectal center and due to referral of many patients from external institutions specifically for EVT, we are able to present data from a large number of consecutive patients treated with EVT for colorectal defects. Therefore, this study reports on effectiveness and safety of EVT for in- and outpatient treatment of colorectal defects. Furthermore, factors predicting therapy success and length of treatment for various indications are analyzed.

## Patients and methods

### Design and study population

**Fig. 1:**
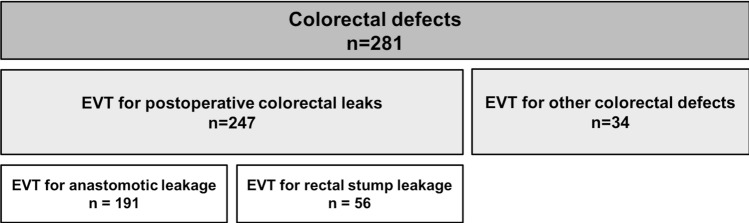
Distribution of EVT for colorectal defects (*N* = 281). Distribution of patients treated with endoscopic vacuum therapy (EVT) for colorectal defects between 2001 and 2019 including 52 patients who had undergone surgery at external institutions and were referred specifically for EVT

Patients’ clinical data were derived from a prospective database that has been maintained since the development and establishment of EVT at our institution in 2001. A retrospective analysis including all patients who had undergone EVT for colorectal defects was conducted. A diagram depicting the study population is shown in Fig. 1. In total, 281 patients treated with EVT for colorectal defects between 2001 and 2019 were included in the study. The study was approved by the Ethics Committee of the Faculty of Medicine, Ludwig-Maximilians-University (LMU), Munich, Germany (approval number 19-728, 28/10/2019). The manuscript preparation was carried out according to the STROBE guidelines.

### Indication and technique

**Fig. 2:**
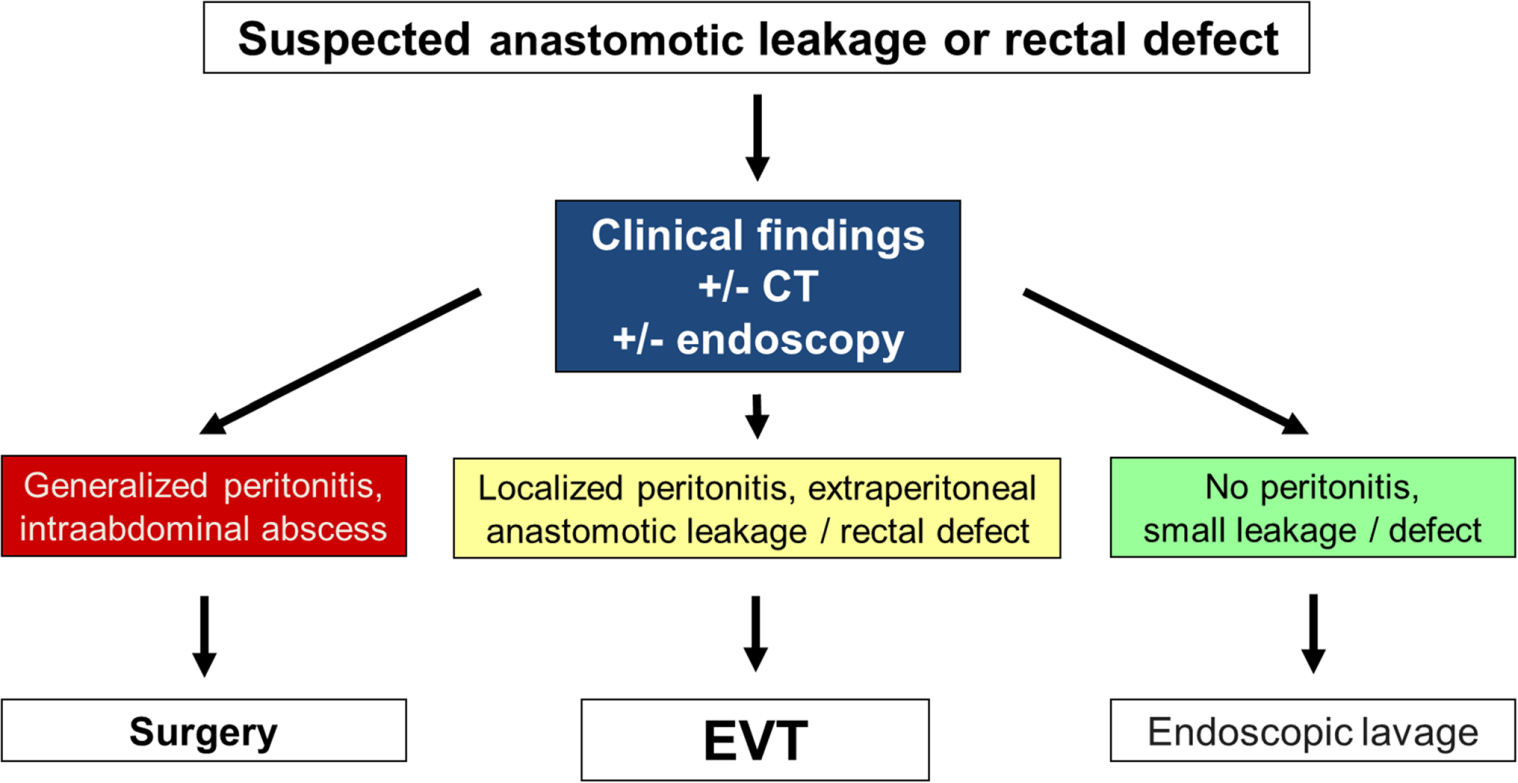
Treatment algorithm in cases with suspected anastomotic leakage or rectal defect (*CT* contrast-enhanced computed tomography, *EVT* endoscopic vacuum therapy)

In general, patients suspicious for a rectal defect after colorectal surgery underwent flexible endoscopy ± computed tomography scan (CT). Postoperative suspicion of rectal leakage was based on clinical and/or laboratory deterioration and drain secretion. Our diagnostic and therapeutic algorithm is illustrated in Fig. 2. EVT was initialized in cases with an extraperitoneal leakage or defect as the primary infectious focus.

EVT was performed as described earlier [1, 4]. In brief, the rectum or its concomitant infectious focus were endoscopically explored. After endoscopic lavage, a polyurethane sponge was placed either directly or by using an overtube device into the infectious cavity (intracavitary) or intraluminal. Intracavitary EVT was performed if the leak size was larger than the diameter of the colonoscope allowing an easy intubation of the cavity. After endoscopic confirmation of correct sponge positioning, a vacuum bottle or pump was connected. Sponge changes were scheduled every 3 days. After successful closure of the wound cavity, the sponge was removed. In case of no local wound improvement or signs of clinical deterioration, EVT was stopped and treatment was adapted to surgery or endoscopic lavage.

### Definition of endpoints

**Fig. 3:**
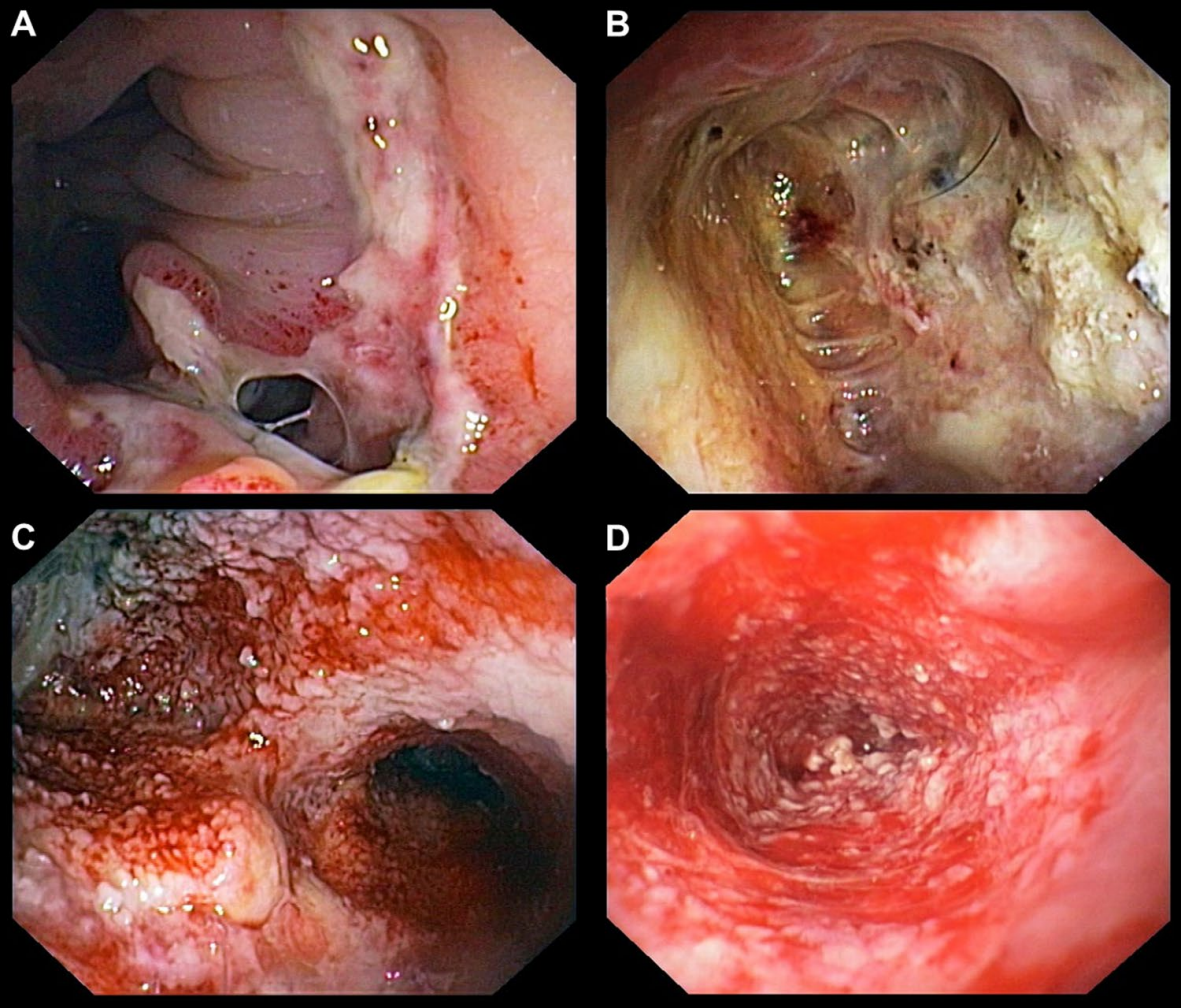
Case showing EVT for treatment of an anastomotic leak after rectal resection. (**A**) Endoscopy in a patient with anastomotic leakage after low anterior resection showing (**B**) the extraperitoneal wound cavity with fibrinous and necrotic tissue. After initiation of endoscopic vacuum therapy (EVT), the cavity becomes clean with increasing granulating tissue (**C**) until EVT can be successfully terminated when the cavity is almost closed with complete granulation (**D**)

Treatment success was assessed during each sponge exchange and was defined as granulating closure of the cavity, more than 90% clean and granulating tissue, decreasing wound secretion, reduction of fibrinous tissue, and no interventional or surgical procedure required in the further course due to local wound healing and successful sepsis control (monitored clinically and by laboratory parameters). Figure 3 shows different stages of wound healing during successful EVT for anastomotic leakage. Figure 3D depicts a status with granulating tissue. At that point we usually stop EVT. EVT was either performed as in- or outpatient treatment. Outpatient treatment was defined as EVT that was conducted mainly or in part in an outpatient setting. Restoration of intestinal continuity (RIC) was defined as the time point when the patient was free of any stoma.

### Statistical analysis

Results were expressed as median and range (minimum and maximum). For evaluation of variables associated with therapy success or outpatient treatment, *χ*^2^-test and Fisher’s exact test (cases of low frequency) or the median test were used where appropriate depending on the variable. For identification of factors associated with therapy duration, a regression model was applied. For analysis of the cumulative odds for RIC analysis, the Kaplan–Meier method was applied. *P* value of <0.05 was considered as statistically significant. For statistical analysis, SPSS statistical software package (version 25, IBM, Chicago, Illinois) was used.

## Results

### Patients’ characteristics

In a total of 281 patients (95 women, 186 men) at a median age of 65 years, colorectal defects were treated with EVT after endoscopic confirmation of diagnosis (Figs. 1, 2). Median time from index operation to the initiation of EVT (TTE) was 10 days (range: 1–91 days). Fifty-two patients had undergone surgery at external institutions and were referred to our endoscopy unit specifically for EVT. The colorectal defect or leakage was symptomatic in all patients and was associated with a relevant clinical or laboratory deterioration. Detailed clinical characteristics of all patients are shown in Table 1.

**Table 1:** Patients’ clinical characteristics

Parameter	*N* (%)
Median age, years (range)	65 (18–96)
Sex	
Female	95 (34)
Male	186 (66)
ASA classification	
2	53 (19)
3	215 (77)
4	13 (5)
Previous chemo-radiation	84 (30)
Previous radiotherapy	11 (4)
Previous chemotherapy	18 (6)
Referred from external hospital for EVT	52 (19)
Underlying disease	
Sigmoid or rectal cancer	183 (65)
Other malignancies (non-CRC)	50 (18)
Diverticular disease	17 (6)
Inflammatory bowel disease	12 (4)
Perforation (traumatic, iatrogenic)	8 (3)
Other benign diseases/tumors	11 (4)
Distant metastases (M1)	38 (14)
Multi-visceral resection	44 (16)
Indication for EVT	
Sigmoid or rectal anastomotic leakage	191 (68)
Rectal stump leakage	56 (20)
Deep APE wound	12 (4)
Rectal fistula ± abscess	11 (4)
Ileo-pouch anal anastomosis	5 (2)
Perforation (traumatic, iatrogenic)	8 (3)
Median time from index operation to the initiation of EVT, days (range)	10 (1–91)
Surgical revision after primary surgery	109 (39)
Surgical revision required at EVT initialization	41 (15)
Median height of anastomotic leak *N* = 191, cm (range)	5.0 (0–12)
Median length of rectal stump *N* = 56, cm (range)	6.5 (2–15)
Median duration of EVT treatment, days (range)	25 (1–258)
Median number of sponge changes (range)	8 (0–64)
Sedation required for sponge changes (*N* = 227)	124 (55)
Outpatient treatment	136 (49)
Morbidity by EVT	5 (2)
Luminal stenosis^a^	16 (6)
Rectal fistula (recto-vaginal)	7 (2)
Bleeding	4 (1)
90-day mortality	5 (2)
EVT successful	256 (91)

^a^Symptomatic with requirement of balloon dilatation

The most common underlying diseases were malignancies. Colorectal cancer (CRC; primary and recurrent sigmoid or rectal cancer, 65% of patients) was the most common diagnosis, followed by pelvic manifestation of other malignant tumors (non-CRC) such as locally advanced ovarian cancer, cancer of the cervix uteri, transitional cell carcinoma, gastrointestinal stroma tumors, neuroendocrine tumors, or uterine sarcoma. In 224 patients (80%), a stoma (proximal fecal diversion) had already been created before or within (protective or permanent) the index operation so that EVT was initiated after fecal diversion. In 37 patients (13%), a stoma was secondarily created at the time of diagnosis of anastomotic leak, whereas in 20 patients (7%, 35% of patients without fecal diversion after the index operation) EVT was initiated without a stoma.

### Treatment duration and EVT-associated morbidity

Median duration of EVT for the entire cohort was 25 days (range: 1–258 days) with a median sponge-changing interval of three days. A median of 8 endoscopic sessions and 7 sponge changes per patient were observed. The duration stratified upon the underlying diagnosis and the indication for EVT and are shown in Tables 2 and 3. Patients with chronic inflammatory bowel disease (IBD) tended to have longer treatments compared to all other patients (40 days vs. 24 days; *P* = 0.118), whereas this was only significant for patients treated for leakage of ileo-pouch anal anastomosis (IPAA) (102 vs. 24 days; *P* = 0.009). EVT for diverticular disease and other benign (non-perforation) indications was observed to be associated with significant shorter treatment periods (13 vs. 27 days; *P* < 0.001). Previous chemo-radiation (*P* = 0.006) and the need for sedation for sponge changes (*P* = 0.006) were significantly associated with longer duration of EVT. Of note, TTE was not linked to longer treatment duration (*P* = 0.156). In addition, no differences were found between patients with and without fecal diversion (*P* = 0.438).

**Table 2:** Duration, success rate, and outpatient treatment of EVT for colorectal defects stratified upon the underlying diagnosis

	Median duration of EVT Days (range)	Therapy success *N*^a^ (%)	Outpatient treatment *N*^a^ (%)
All patients (*N* = 281)	25 (1–258)	256 (91)	136 (49)
Sigmoid or rectal cancer (*N* = 183)	27(1–223)	170 (93)	96 (52)
Other malignancies (no-CRC; *N* = 50)	23 (2–258)	43 (86)	23 (46)
Diverticular disease (*N* = 17)	17 (6–56)	14 (82)	6 (35)
Inflammatory bowel disease (IBD, *N* = 12)	40 (13–151)	12 (100)	4 (12)
Perforation (traumatic, iatrogenic; *N* = 8)	21 (9–105)	7 (88)	3 (38)
Other benign diseases/tumors (*N* = 11)	12 (6–46)	10 (91)	4 (36)

^a^Percentages of the respective subgroup patient number

**Table 3:** Duration, success rate, and outpatient treatment of EVT for colorectal defects stratified upon the indication for EVT

	Median duration of EVT Days (range)	Therapy success *N*^a^ (%)	Outpatient treatment *N*^a^ (%)
All patients (*N* = 281)	25 (1–258)	256 (91)	136 (49)
Sigmoid or rectal anastomotic leakage (*N* = 189)	26 (1–258)	176 (93)	98 (52)
Rectal stump leakage (*N* = 56)	20 (7–189)	47 (84)	25 (45)
Deep APE wound (*N* = 12)	37 (6–223)	12 (100)	3 (25)
Rectal fistula ± abscess (*N* = 11)	21 (2–62)	10 (91)	7 (64)
Ileo-pouch anal anastomosis leakage (*N* = 5)	102 (42–151)	5 (100)	1 (20)

^a^Percentages of the respective subgroup patient number

The subgroup analysis of patients undergoing EVT for anastomotic leaks revealed preoperative radiotherapy or chemoradiotherapy (*P* = 0.013), performing a total mesorectal excision (compared to partial mesorectal excision; *P* = 0.043), and a malignant diagnosis as indication for the index operation (*P* = 0.022) as predictive factors for elongated therapy duration.

As EVT-associated morbidity, symptomatic luminal stenosis occurred in 16 (6%) patients and had to be treated by endoscopic balloon dilation. Rectal fistulas occurred in 7 patients (2%) after a median of 22 days after EVT initiation. All fistulas were recto-vaginal, and in the majority of these patients, initial surgery had involved resection of parts of the vagina or the uterus. In four patients (1%), EVT-associated lower gastrointestinal bleeding occurred which could be controlled endoscopically in three and surgically in one patient.

### Treatment success and follow-up

In 256 patients (91%), EVT was successful and local control of inflammatory focus was achieved. The main reason for EVT failure (*N* = 25) was insufficient granulation with persistent sepsis (*N* = 18, 72%). The majority of these patients required redo surgery. Other reasons for EVT failure were mortality (*N* = 5, 20%) and patient wish (*N* = 2, 8%).

The success rate depending on the underlying diagnosis and the indication for EVT is shown in Tables 2 and 3. EVT following multi-visceral resection (*P* = 0.037), recent surgical revision after primary surgery (*P* = 0.009), and the duration of EVT treatment (*P* = 0.001) were associated with unsuccessful treatment. Being referred from external hospital for EVT (*P* = 0.097) tended to predict therapy failure, however, TTE was not associated with unsuccessful EVT (*P* = 0.871). Furthermore, we did not find differences between patients with and without fecal diversion. The success rates were 91%, 95%, and 90% in patients with primary, secondary, and without fecal diversion (*P* = 0.723). Failure of EVT was recognized rather early after a median of 9 days, on average after the third sponge exchange. Time of diagnosis or start of EVT was not associated with success or length of treatment. The possibility of outpatient treatment (success rate 98% vs. 88%; *P* = 0.025) was significantly associated with therapy success in patients with anastomotic leak.

**Fig. 4:**
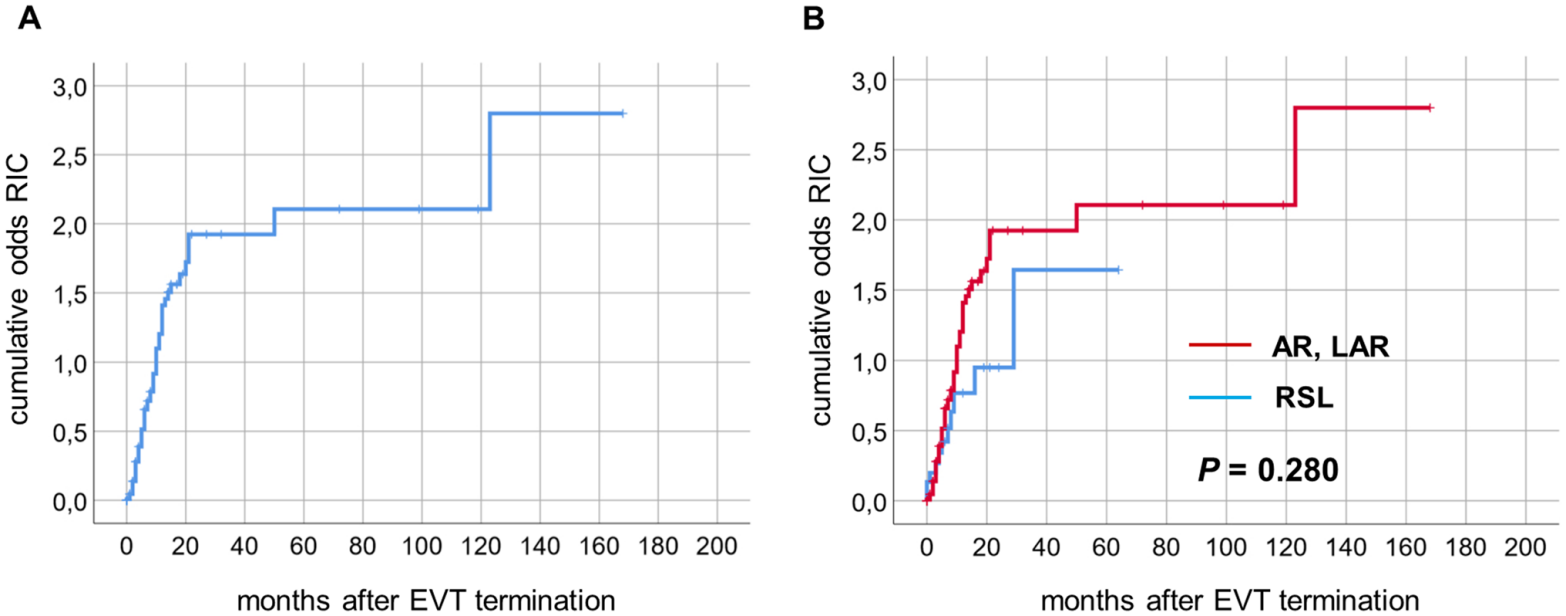
Restoration of intestinal continuity. **A** Cumulative odds for restoration of intestinal continuity (RIC) after EVT for colorectal defects. **B** Comparing the odds of patients undergoing EVT for anastomotic leakage following anterior (AR) and low anterior resection (LAR) with rectal stump leakage (RSL) where technically possible

Restoration of intestinal continuity (RIC) was generally not possible in 60 patients (21%) due to early-phase mortality, performance of abdominoperineal extirpation, or oncological reasons such as extensive metastatic disease. In 132 out of 221 patients (60%) with possible restoration, RIC was achieved in the further course within a median follow-up of 6 months. In these patients, median time to intestinal continuity was 9 months with a 12-month RIC rate of 52% (Fig. 4A). When comparing EVT for sigmoid or rectal anastomotic leakage and rectal stump leakage in patients in whom RIC was technically possible (*N* = 176 and *N* = 24, respectively), no significant differences were observed regarding reversal rates (overall rate, 63% vs. 50%) and time to RIC (9 months, respectively, *P* = 0.448). When analyzing patients with sigmoid or rectal resections for colorectal cancer without multi-visceral resections (*N* = 124), RIC rate revealed to be 68% overall within a median follow-up of 7 months. This tended to be better compared to patients with rectal stump leakage (*P* = 0.280, Fig. 4B).

### Outpatient treatment

Primary or secondary outpatient treatment was possible in 136 patients (49%). Primary (complete) outpatient EVT was conducted in 5 patients with a median duration of 31 days (range: 13–56) and a success rate of 100%. Secondary outpatient treatment (continued EVT after discharge) led to a median reduction of 15 days (range: 1–364) of the hospital stay which corresponded to 46% of the total EVT duration in these patients. Success rate of secondary outpatient treatment was 98%. Outpatient treatment stratified upon underlying diagnosis and treatment is shown in Tables 2 and 3. Younger patient age (*P* = 0.044) and duration of treatment (*P* < 0.001) were factors that were found to be significantly associated with the possibility of outpatient treatment.

## Discussion

Postoperative, traumatic, or iatrogenic colorectal leaks are associated with a significant increase in morbidity and mortality [8, 9], representing a challenging situation for both patient and treating physician. The reported incidence of postoperative anastomotic leakage ranges between 6 and 30% with an average of 11%, depending on the height of the anastomosis [9, 10]. Redo operations such as Hartmann’s procedure are high-risk interventions with relevant mortality and low rates of RIC [11–13]. Therefore, a safe, effective, and well-validated minimally invasive technique is urgently needed in order to attain the best possible short- and long-term outcomes. Even though data are still scarce, EVT has become the most common endoscopic technique for treatment of colorectal leaks after rectal resection [5], and currently, commercial systems are distributed in more than 40 countries worldwide [5, 6]. Compared to stent application or fibrin glue, EVT appears to be a much more versatile endoscopic technique because it allows for the treatment of defects in almost all extraperitoneal locations, regardless of location or size of the defect. In contrast, the usage of stents entails limitations that include patient discomfort and stent migration, hence, stent insertion should be avoided in lower rectal defects. Furthermore, stents can only be used for small abscess cavities [14]. In the case of a larger abscess cavity, an additional percutaneous drainage needs to be placed; in contrast to EVT, stents do not allow internal drainage. Similarly, the use of fibrin glue comes with a limitation that allows it to be used only on very small leaks without any cavity or abscess behind [15].

A recently published review has analyzed the available data on EVT for colorectal defects [7]. Analyzed data in that review were derived from 17 different studies/case series, comprising 276 patients in total. Besides having a small sample size, included case series are characterized by a strong clinical heterogeneity, caused by the use of different materials, methods, treatment algorithms, and indications (e.g., no differentiation between Hartmann stump and anastomotic leakage after rectal resection). Despite a large range in treatment success (56–97%), there was a weighted mean success rate of 85.3% among all included studies which is similar to the success rate of 91% in our large patient cohort. Some factors influencing success or failure of EVT have been identified. In contrast to some other studies [7], neoadjuvant chemo-radiation was not linked to EVT failure but to the requirement for longer treatment duration. In addition, we identified additional risk factors for EVT failure such as multi-visceral resections and recent surgical revision after primary surgery. Of note, the time from index operation to initiation of EVT had no influence on therapy success. Our technical experience is that older or chronic leaks are stiffer but respond well to EVT after thorough endoscopic lavage and curettage with an endoscopic brush. We also succeeded in several patients without fecal diversion. In patients with rectal anastomosis, in whom no protective stoma had been created before the diagnosis of anastomotic leakage, 20 patients were selected for EVT without secondary fecal diversion. Requirements for considering EVT without fecal diversion are the possibility of complete intracavitary sponge placement with complete sealing towards the lumen and sufficient anal sphincter function for maintaining negative pressure. This approach was successful in 90% of the selected patients.

Recently, we have published detailed results of EVT for rectal stump leakage. EVT was conducted as intracavitary or intraluminal treatment with a success rate of 84% [16]. Preoperative radiation was shown significantly associated with EVT failure, and patient age represented a predictive factor for therapy duration [16].

Despite these promising results in the literature, there is currently only little evidence that EVT might be superior to “conventional” treatment for anastomotic leakage. According to a recently published small comparative study [6], EVT might be more effective than conventional treatment with regard to definite healing of postoperative leaks and long-term preservation of intestinal continuity [6]. Here, EVT was associated with long-term preservation/restoration of intestinal continuity in 87% compared to 38% of patients who had received conventional treatment. These numbers are in agreement with the existing literature where stoma reversal after leakage is performed in 30–50% of patients [17, 18], compared to a weighted mean rate of 76% in patients across studies using EVT [7].

According to our analysis, EVT appears to be a safe and well-tolerated procedure. In line with other studies [7], luminal stenosis (6%) is the most frequent adverse event. All stenoses were successfully treated with balloon dilatation. Of note, anastomotic stenoses also occur due to chronic inflammation in patients who did not receive EVT and may be caused by the anastomotic leakage itself rather than by EVT [19]. In contrast to other studies [7], we observed very few EVT-induced recto-vaginal fistulas as EVT was strictly used for extraperitoneal defects only. Fistulas occurred after a median EVT duration of 22 days and in the majority of these patients, initial surgery had involved the vagina or the uterus suggesting that EVT might have prompted or revealed a leak at the vagina either.

The long duration of therapy is one of the major concerns regarding EVT, and a median treatment duration of 47 days—as calculated among 17 studies [7]—is, indeed, hard to justify. Another review found a median treatment duration of 31 days among 19 studies [20]. However, the median number of patients in the included studies in these reviews was only fifteen [7, 20]. This extensive treatment length with this technique might partially be explained by a lack of experience in the various working groups. For physicians who have limited experience with EVT, it would be a challenge to determine the correct EVT duration and termination. A timely decision needs to be made to stop EVT treatment, either because of sufficient or insufficient wound healing. In our cohort, median treatment duration of EVT was 25 days, but this is reduced to 17 days in benign diseases such as diverticular disease. Unsuccessful EVT was noted in 68% and 84% of failure patients during the first 14 and 21 days, respectively, and treatment was adapted accordingly. Consistent with the available literature, treatment duration was affected by neoadjuvant chemo-radiation. In addition, our analysis revealed that the type of underlying disease and the indication for EVT is linked to its duration. According to Van Koperen et al. [21], the timing of EVT can additionally influence treatment success, with a success rate of 75% (6 of 8 patients) when EVT was commenced within 6 weeks after initial surgery, compared to 38% (3 of 8) when started more than 6 weeks after the initial surgery [21]. Besides a trend in our analysis, however, we could not clearly confirm this finding. Of note, patients referred from external institutions tended to have a lower success rate.

In the current study, we have further demonstrated that EVT can be conducted effectively as an ambulatory treatment for eligible patients with a foreseeably longer treatment duration. Through ambulantization, the length of hospital stay was reduced by a median of 15 days. Success rates of EVT were 100% and 98% for primary and secondary outpatient treatment, respectively. Ambulatory vacuum-assisted wound therapy has been successfully conducted and described for other indications such as diabetic foot ulcers [22]. Although ambulatory EVT has not been described in detail before, it appears to be safe and well tolerated by the patients. As treatment duration seems to be associated with certain risk factors such as chemo-radiation, ambulatory EVT as a treatment option should be discussed early especially when treating such patients. Besides the monetary aspects, the patients’ quality of life is also possibly improved by allowing treatment in surroundings preferable for the patient.

In this first larger cohort study, EVT was shown to be a safe and effective treatment option for colorectal leaks and perforations. EVT might become increasingly recognized as an ambulatory treatment option reducing the length of hospital stay.
